# Pharmacoepigenetics in type 2 diabetes: is it clinically relevant?

**DOI:** 10.1007/s00125-022-05681-x

**Published:** 2022-03-21

**Authors:** Charlotte Ling

**Affiliations:** Epigenetics and Diabetes Unit, Department of Clinical Sciences, Lund University Diabetes Centre, Lund University, Scania University Hospital, Malmö, Sweden

**Keywords:** Adipose tissue, Beta cells, Blood, Blood-based epigenetic biomarkers, DNA methylation, Drug targets, Epigenetic enzymes, Epigenetics, Histone modification, Inhibitors, Liver, Non-coding RNA, Pancreatic islets, Pharmacogenetics, Precision medicine, Skeletal muscle

## Abstract

Data generated over nearly two decades clearly demonstrate the importance of epigenetic modifications and mechanisms in the pathogenesis of type 2 diabetes. However, the role of pharmacoepigenetics in type 2 diabetes is less well established. The field of pharmacoepigenetics covers epigenetic biomarkers that predict response to therapy, therapy-induced epigenetic alterations as well as epigenetic therapies including inhibitors of epigenetic enzymes. Not all individuals with type 2 diabetes respond to glucose-lowering therapies in the same way, and there is therefore a need for clinically useful biomarkers that discriminate responders from non-responders. Blood-based epigenetic biomarkers may be useful for this purpose. There is also a need for a better understanding of whether existing glucose-lowering therapies exert their function partly through therapy-induced epigenetic alterations. Finally, epigenetic enzymes may be drug targets for type 2 diabetes. Here, I discuss whether pharmacoepigenetics is clinically relevant for type 2 diabetes based on studies addressing this topic.

**Supplementary Information:**

The online version contains supplementary material available at 10.1007/s00125-022-05681-x.

Research studies performed over the last two decades have identified epigenetic modifications and mechanisms that seem to play a role in the pathogenesis of type 2 diabetes [1–18]. The epigenome includes DNA methylation, histone modifications and non-coding RNA [1]. There are epigenetic modifications which are stable over time and those that change due to short-term and/or long-term environmental exposures such as drugs, diet, exercise or stress, as well as ageing [19–25]. However, more work is needed before we fully understand the environmental regulation of the epigenome in all human cell types. Moreover, although numerous studies have investigated the role of pharmacogenetics in type 2 diabetes [26–31], the interest in pharmacoepigenetics has been limited [32]. So, what is the definition of pharmacoepigenetics and is it clinically relevant for type 2 diabetes? The meaning of pharmacoepigenetics is not set in stone but can be divided into: (1) blood-based epigenetic biomarkers that predict response or tolerance to therapy; (2) individual differences in response to therapy due to epigenetic mechanisms or variation in target cells and tissues; (3) therapies that alter the epigenome or epigenetic mechanisms, which subsequently may contribute to their effect; and (4) epigenetic therapies (Fig. 1). Below, I discuss some studies addressing these points in relation to type 2 diabetes.

**Fig. 1:**
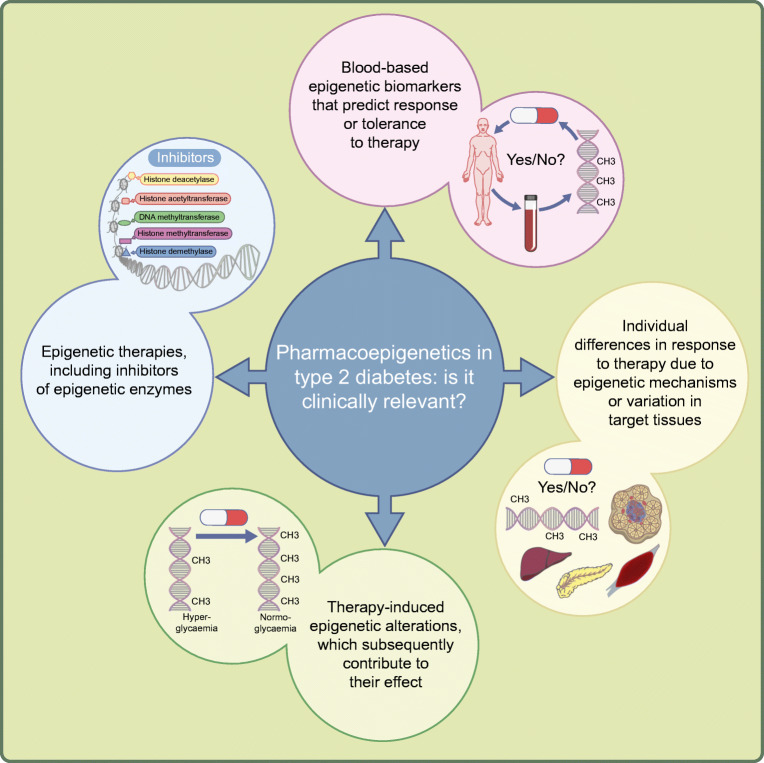
Pharmacoepigenetics in type 2 diabetes. The figure shows different aspects of pharmacoepigenetics that could be applied to type 2 diabetes prediction, response and treatment strategies. This figure is available as a downloadable slide

It is well established that all individuals do not respond to therapies in the same way. For example, ~30% of individuals with type 2 diabetes do not have a glucose-lowering response to metformin, and ~5% suffer from intolerable side effects, including gastrointestinal problems [33, 34]. Currently, there are no clinically useful biomarkers that predict response and tolerance to metformin. Nevertheless, a recent study supports the use of blood-based epigenetic biomarkers for prediction of glycaemic response and intolerance to metformin in newly diagnosed individuals with type 2 diabetes [32]. Here, increased DNA methylation of 11 CpG sites in the blood was associated with a higher risk of not responding to metformin, and increased methylation of four other CpG sites was associated with a higher risk of not tolerating metformin in drug-naive newly diagnosed individuals with type 2 diabetes. Methylation risk scores (MRS) generated based on DNA methylation levels of these 11 and four sites could clearly discriminate glycaemic responders from non-responders, and tolerant from intolerant patients to metformin therapy in three different cohorts. These results promote the further development and future use of blood-based epigenetic biomarkers for precision medicine in type 2 diabetes (Fig. 1). Therefore, pharmacoepigenetics seem to be clinically relevant for type 2 diabetes. Other factors, for example genetic variation, clinical phenotypes and gut microbiota, should further be explored, and combinations of different phenotypes may ultimately generate scores with the best predictive capacity for response to glucose-lowering therapies [26–29, 35–37]. Of note, in the field of cancer, both epigenetic biomarkers in blood and tissues have proven to be clinically relevant [38]. However, individual differences in response to pharmacotherapy due to epigenetic mechanisms and modifications in target tissues are, to my knowledge, not well studied in type 2 diabetes, but could be important (Fig. 1). Such epigenetic mechanisms may include DNA methylation and/or histone modifications of drug transporters, affecting the levels of these transporters in target cells and hence their ability to take up or excrete drugs.

Therapy-induced epigenetic alterations may be clinically relevant and may benefit patients (Fig. 1). Pharmacotherapies currently used for lowering blood glucose and for treatment of lipid dysregulation can alter the epigenome in tissues and cells from patients with type 2 diabetes and individuals without diabetes [25, 39–42]. For instance, individuals with type 2 diabetes who took metformin had altered DNA methylation of genes encoding the metformin transporters OCT1, OCT3 and MATE1 in the liver compared with those who did not receive any medication [39]. Short-term metformin exposure also altered DNA methylation in the blood of individuals without diabetes [25]. Additionally, incretin drugs, e.g. GLP1R agonists, prevented glucose-induced reductions in DNA methylation of *NFKB1* and *SOD2* in human aortic endothelial cells, which may affect vascular complications [40]. Incretin treatment was also shown to reverse epigenetic modifications associated with diabetes in rodents exposed to an impaired intrauterine environment [43]. Statin therapy was recently associated with differential DNA methylation in blood from individuals with type 2 diabetes as well as in individuals without diabetes [41, 42]. These include differential methylation of sites annotated to *ABCG1*, *DHCR24* and *SC4MOL* (also known as *MSMO1*), which encode proteins involved in the transport and biosynthesis of cholesterol. Causal mediation analyses further suggest that DNA methylation may mediate some of statin’s effects on metabolic phenotypes [41, 42]. Overall, pharmacotherapies used for treatment of type 2 diabetes and lipid dysregulation can induce epigenetic modifications in human cells (Fig. 1). Nevertheless, further work is needed before concluding the clinical benefits or disadvantages of therapy-induced epigenetic modifications in individuals with type 2 diabetes.

Finally, can epigenetic therapies be used for treatment of type 2 diabetes (Fig. 1)? And what are epigenetic therapies? Inhibitors of epigenetic enzymes, such as DNA methyltransferase (DNMT) and histone deacetylase (HDAC) inhibitors, fall into the category of epigenetic therapies, and such drugs are currently in use, or in clinical trials, for treatment of different cancers [44]. Interestingly, epigenetic enzymes were found to be dysregulated in cells and tissues from individuals with type 2 diabetes compared with individuals without type 2 diabetes, as well as in cells exposed to diabetogenic conditions, suggesting a potential role for epigenetic therapies also in diabetes [5, 6, 9–11, 45]. For example, individuals with type 2 diabetes had higher DNMT3B levels in cultured myotubes [5] and decreased TET1 expression in adipose tissue [6] vs tissue from individuals without type 2 diabetes, while palmitate exposure decreased *DNMT3A* and *DNMT1* expression in human pancreatic islets [11]. Several studies have further shown that inhibitors of HDACs and histone demethylases, or silencing and overexpressing those enzymes, impact beta cell function and insulin secretion [9, 46–49]. For example, DNA methylation is decreased, and expression of *HDAC7* increased in pancreatic islets from donors with type 2 diabetes [49]. Overexpression of *Hdac7* in clonal beta cells and rat islets impaired glucose-stimulated insulin secretion, while exposure to two different HDAC inhibitors, trichostatin A (TSA) and MC1568, reversed the negative effect of *Hdac7* overexpression on insulin secretion and mitochondrial function [9, 49]. MC1568 also increased glucose-stimulated insulin secretion in pancreatic islets from donors with type 2 diabetes cultured in vitro [49]. Other studies investigating the impact of the inhibition of epigenetic enzymes in muscle, adipose tissue and liver found improved metabolism and cell function [50–52]. However, the chronic nature of type 2 diabetes results in long-term use of therapies. It is therefore important to weigh benefits against risks, and global action of inhibitors of epigenetic enzymes may lead to intolerable side effects. More selective inhibitors and/or cell-specific delivery may represent avenues for future therapeutic purposes.

Altogether, existing literature suggests that pharmacoepigenetics may be clinically relevant for type 2 diabetes. But there is still a lot of work needed before pharmacoepigenetics in any of the research areas mentioned above may reach the clinic and help individuals with type 2 diabetes receive optimal treatment, reducing their complications and suffering.

## Supplementary information

ESM 1 (PPTX 350 kb)

## Abbreviations

DNMT: DNA methyltransferase
HDAC: Histone deacetylase
