# Supplementary material for: Pharmacoepigenetics in type 2 diabetes: is it clinically relevant?
Source: Diabetologia. 2022 Mar 21;65(11):1849–53. doi: 10.1007/s00125-022-05681-x (PMC9522755; doi:10.1007/s00125-022-05681-x)
Supplement: Supplementary file 1 — (PPTX 350 kb) [file 125_2022_5681_MOESM1_ESM.pptx]

## Slide 1
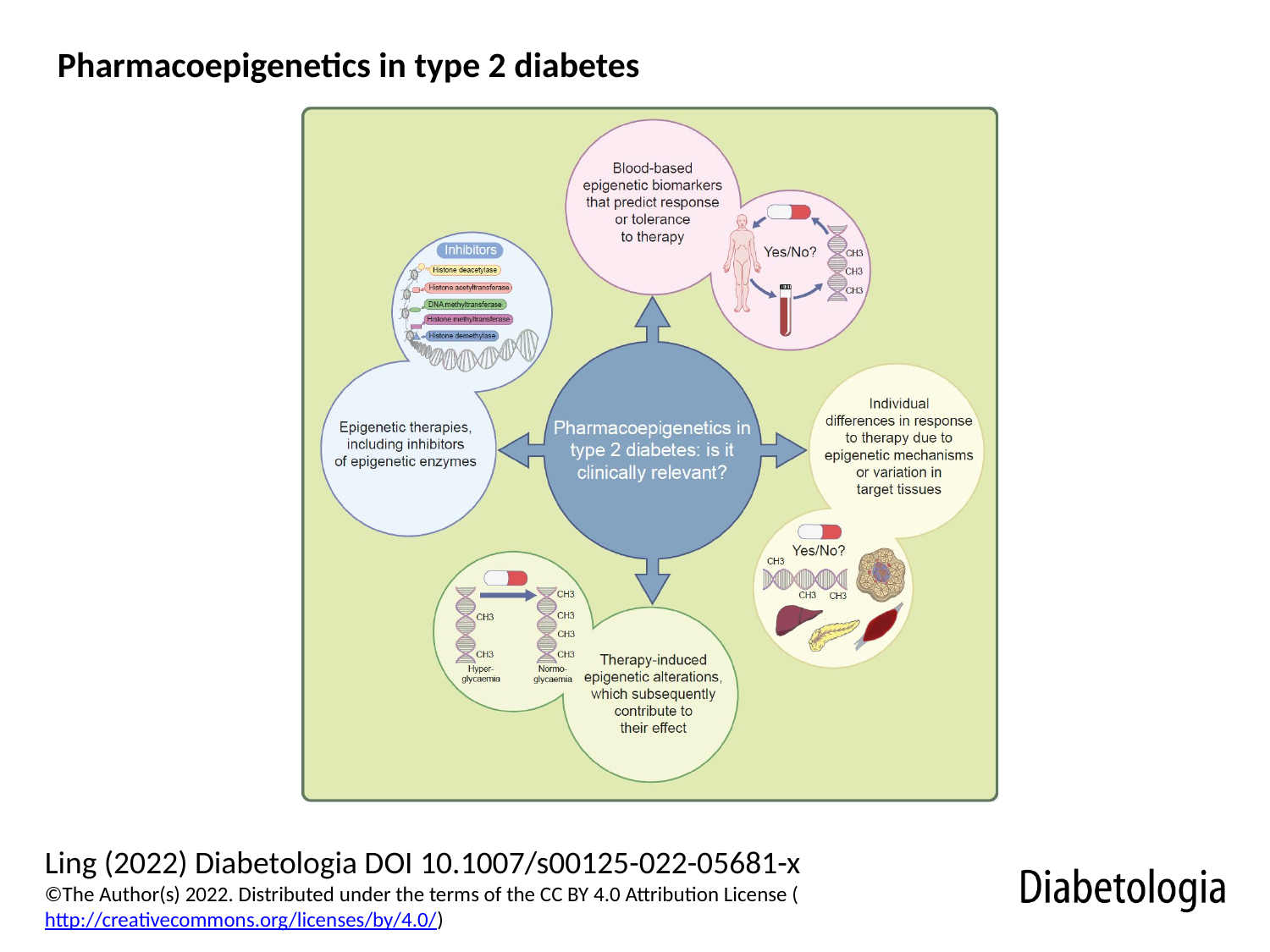

Pharmacoepigenetics in type 2 diabetes
Ling (2022) Diabetologia DOI 10.1007/s00125-022-05681-x
©The Author(s) 2022. Distributed under the terms of the CC BY 4.0 Attribution License (http://creativecommons.org/licenses/by/4.0/)
